# Effect Of Hypo−and Euthyroid Status On Serum Cystatin C Levels

**DOI:** 10.4274/jcrpe.v2i4.155

**Published:** 2010-11-04

**Authors:** Tülin Ayşe Özden, Hüseyin Tekerek, Firdevs Baş, Feyza Darendeliler

**Affiliations:** 1 İstanbul University İstanbul Faculty of Medicine, Department of Pediatrics, Pediatric Gastroenterolgy−Hepatology and Nutrition Unit, İstanbul, Turkey; 2 İstanbul University, İstanbul Faculty of Medicine, Department of Pediatrics, İstanbul, Turkey; 3 İstanbul University, İstanbul Faculty of Medicine, Department of Pediatrics, Pediatric Endocrinology Unit, İstanbul, Turkey; +90 212 532 42 33+90 212 533 13 83feyzad@istanbul.edu.trIstanbul University, Faculty of Medicine, Department of Pediatrics, Pediatric Endocrinology Unit, İstanbul, Turkey

**Keywords:** Thyroid dysfunction, hypothyroidism, Hyperthyroidism, cystatin C, renal function

## Abstract

**Objective**: The aim of this study was to investigate the effect of hypo− and euthyroid status on serum cystatin C (CysC) levels in children and to explore whether CysC can be used as a marker of the thyroid status.

**Methods**: Twenty five patients with hypothyroidism (10M, 15F; mean age:8.7±4.9 years) and 21 healhty age−matched controls (9M, 12F; mean age: 9.7±5.0 years) were included in this study. Serum thyrotropin (TSH), free thyroxine (fT4), serum CysC and creatinine levels were studied in patients with hypothyroidism twice, i.e. in the euthyroid (on L−T4) and hypothyroid state, and in controls.

**Results**: No significant differences in creatinine, glomerular filtration rate (GFR) and CysC levels were observed between the study group in the euthyroid status and the control group. CysC (mg/dL) level was found to be lower in the hypothyroid status(0.6±0.1) than in the euthyroid status (0.66±0.1) (p=0.01). In hypothyroid status, CysC levels showed a positive correlation with GFR (r=0.463, p=0.02) and GFR had positive

correlation with fT4 (r=0.563, p=0.012).

**Conclusions**: We demonstrated a significant effect of thyroid dysfunction on CysC levels, but the changes in serum CysC levels in hypo− and euthyroid status did not exceed the reference interval. It may be concluded that serum CysC levels have limited use in evaluating the peripheral effects of thyroid hormones.

**Conflict of interest:**None declared.

## INTRODUCTION

Cystatin C (CysC) is a low−molecular−weight (MW 13360), nonglycocylated cationic basic protein consisting of 120 amino acids and belongs to the cystatin superfamily of endogenous cysteine proteinase inhibitors (1, 2, 3, 4). Although serum creatinine concentration is the most commonly used indicator of kidney function, it is not an accurate marker of glomerular filtration rate (GFR), which is an important index of renal function in health and disease. On the other hand, the plasma concentration of CysC has been shown to be a better marker of GFR in both adults and children (5, 6, 7). CysC possesses most of the properties of an ideal GFR test − produced by all nucleated cells at a constant rate, it is freely filtered from the glomerulus and finally, is fully destroyed in the proximal renal tubulus (5, 6, 7). Its rate of production is not influenced by inflammation or malignancy and, unlike creatinine, is unaffected by muscle mass, gender, or age (8, 9, 10). The levels are constant from the age of 1 year onwards (8). CysC might offer a considerable advantage in the measurement of GFR (6).

Thyroid hormones, through their general metabolic effects, may also influence plasma CysC levels. There are limited studies on serum CysC levels in thyroid diseases. Thyroid function has an impact on both CysC and creatinine levels. Serum CysC levels have been found to be low in hypothyroidism and to increase when a euthyroid status is attained. Serum creatinine levels have been demonstrated to be relatively high in hypothyroidism and low in hyperthyroidism when compared to the euthyroid state. These CysC alterations may be due to changes in the synthesis of CysC, but could also be due to changes in clearance, as suggested for creatinine (11, 12, 13, 14, 15, 16, 17, 18).

The objectives of this study were to evaluate the effects of thyroid hormone status on CysC levels as well as to explore whether CysC levels can be used as a marker of peripheral effects of thyroid hormones, as may be needed in cases of thyroid hormone resistance.

## METHODS

A total of 25 patients with permanent congenital hypothyroidism (thyroid aplasia, n=6; ectopic thyroid, n=11; dyshormonogenesis, n=8) were included in the study group. No evidence of accompaning disease was observed in any of the children. Twenty−one age−matched healthy children formed the control group.

At the time of investigation, weight and height measurements were taken by an auxologist using standard methods and techniques and were expressed as standard deviation score (SDS) according to national standards (19). Body mass index (BMI) was calculated as kg/m2 and also expressed as SDS using national standards (20). The anthropometric data of the two groups are given in Table 1.

Serum thyrotropin (TSH), free thyroxine (fT4), serum CysC and creatinine levels were studied in patients with hypothyroidism twice − in euthyroid and hypothyroid states. Euthyroid status was defined when patients on L−T4 therapy had normal fT4 and decreased TSH levels compared to the levels at onset. The children were accepted to be in a hypothyroid status when they did not receive L−T4 therapy and had abnormal fT4 and TSH levels. Hypothyroid levels were obtained either at the time of diagnosis of hypothyroidism before initiation of therapy or after stopping L−T4 therapy for 4 weeks in those already receiving therapy. The L−T4 therapy was discontinued in some patients, because we wanted to search the underlying etiology in these patients. These patients had been started on therapy at diagnosis and no tests had been done with respect to etiology at that time. Thus, we had an opportunity to study CysC levels during the period off L−T4 therapy.

Blood samples were taken in the morning in the fasting state and in patients on therapy, before the L−T4 dose. Blood samples were also taken from the children in the control group in the morning for determination of serum fT4, TSH, CysC and creatinine levels. Blood samples were immediately centrifuged and deep frozen at −20°C until analyses. Serum creatinine was measured on the same day.

FT4 (pmol/L) was determined by [125I] RIA kit (Institute of Isotopes Ltd., Budapest, Hungary) and TSH (mU/L) was measured by IRMA using DSL−5300 kit (Diagnostic Systems Laboratories, Texas, USA). Serum CysC was analyzed by particle−enhanced immunoturbidimetry (21) using the CysC PET−kit ( DAKO, Hamburg, Germany) by auto−analyzer (Cobas−Mira Plus) and serum creatinine was analyzed by Jaffe method on a Cobas Integra 800 analyzer (Roche Diagnostics, Mannheim, Germany). Measuring range of CysC assay was approximately 0.4−7.5 mg/L. The limit of detection was 0.07 mg/L. Total assay coefficient of variation (CV) was 2.0−5.9 %. Reference intervals for serum CysC, creatinine, TSH and fT4 were taken as 0.55− 1.15 mg/L, <1mg/dL, 0.3−4.2 mIU/L and 12−22 pmol/L, respectively, according to the reference ranges given in the kits. GFR was calculated using the Schwartz formula [k (constant) x height (cm)/ serum creatinine] (22).

The study was approved by the local ethics committee. Written informed consent was obtained from the parents of the children in both the study group and the control group.

**Statistical Analysis**

Statistical analysis was done using the SPSS−PC (SSPS for Windows, version 12.0, Chicago) package program. Mean (±SD) values are given. Between−group comparisons were done by the Mann−Whitney U test and t−test and within−group comparisons were performed by the Wilcoxon signed−rank test and paired t−test. Pearson’s correlation coefficient was applied to detect linear correlation in the total group. A p−value of less than 0.05 was considered statistically significant.

**Table 1:**
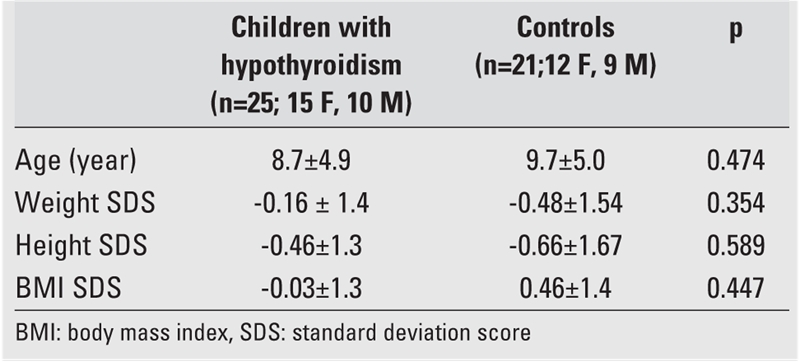
Anthropometric parameters of the children with hypothyroidism and of the controls

## RESULTS

At the time of investigation, the mean age of the children with hypothyroidism was 8.7±4.9 years (range:2.9−19.1 years) and was not significantly different from the age of the control group (9.7±5.0 years; range: 1.5−19.8 years). As seen in Table 1, anthropometric parameters of the two groups were not different from each other either. Serum fT4 (pmol/L) was 4.6±4.4 (range:0.1−12.67) and serum TSH (mIU/L) was 201.8 ± 228.3 (range:11.53−954.1) in the hypothroid status and fT4 was 20.6±3.8 (range:16.6−34.6) and TSH was 4.7±2.6 (range:0.3−10.8) in the euthyroid satus in children with primary hypothyroidism. In some children, TSH values showed a decrease, but were still high at the time of investigation, while fT4 was normal, indicating a euthyroid status.Control group had normal fT4 (pmol/L) and TSH (mIU/L) values [17.7±2.1 (range: 13.5−22.2) and 3.1±1.1 (range:1.6−5.5), respectively], fT4 and TSH values were in normal ranges.

As seen in Table 2, creatinine, GFR and CysC levels did not differ significantly between the study group in the euthyroid status and the control group. Comparison between the hypo− and euthyroid states in the study group demonstrated higher creatinine level (p=0.007), lower GFR (p=0.023) and lower CysC (p=0.01) in the hypothyroid status than in the euthyroid status.

Comparison between the study group in the hypothyroid status and the control group revealed significant differences in the GFR (p=0.002) and in the CysC (p=0.017) levels, but the differences were not significant between the control group and the patients in the euthyroid status.

In the study group, serum CysC levels were not significant difference between boys and girls before treatment (0.64±0.1 vs 0.58±0.1 p=0.113), but were significantly higher in boys than in girls after treatment (0.74±0.1 vs 0.6±0.1, p=0.008).

In controls, serum CysC did not show a significant difference between boys and girls (0.66±0.1 vs 0.68±0.1, p=0.862).

**Correlation Studies**

There was a positive correlation between CysC and GFR in hypothyroid status (r=0.463, p=0.02). CysC level was not correlated to height, weight, BMI and age in both the study and the control group. GFR had positive correlation with fT4 in the hypothyroid status (r=0.563, p=0.012).

**Table 1:**
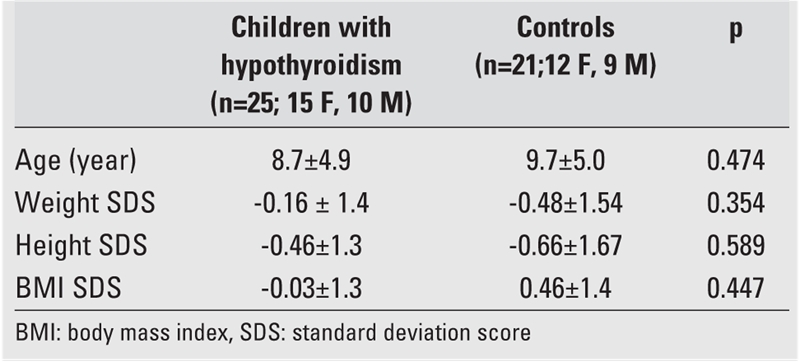
Anthropometric parameters of the children with hypothyroidism and of the controls

**Table 2:**
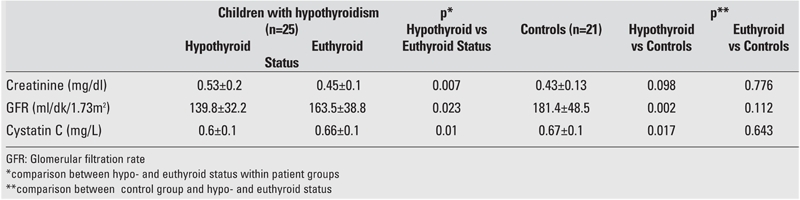
Comparison of some laboratory findings between the study group (Group 1) and the control group (Group 2) (Mean±SD)

## DISCUSSION

The main finding of our study was that serum creatinine was higher, while GFR and CysC levels were lower in hypothyroid status when compared to the euthyroid status and/or to normal controls. These results are consistent with those of most of the studies investigating the effect of thyroid status on serum CysC levels (11, 12, 13, 14, 15, 16, 17, 18, 23). Our study was different from the previous studies in that it was undertaken in children with hypothyroidism, whereas most of the studies were conducted in adults. Secondly, some of the previous studies included patients with hypothyroidism of autoimmune etiology such as autoimmune thyroiditis or Graves’ disease (11, 12, 13, 14, 15, 16, 17, 18) and, it was speculated that autoimmune mechanisms might also play a role in the effects of thyroid dysfunction on CysC serum levels (1). Our study did not include patients with autoimmune etiology, thus the effect of thyroid status on CysC levels may be considered to be the result of hypothyroid status per se.

Thyroid hormones have significant effects on renal hemodynamics, renal handling of salt and water and on the active tubular transport of Na+, K+ and H+ (23). It is possible that tubular creatinine secretion is diminished in hypothyroidism, thereby increasing the serum creatinine concentrations. A slight decrease in GFR has been found in patients with hypothyroidism, which improved significantly after treatment (11, 13, 14). Thyroid hormones may influence CysC levels by altering their production rate and/or their metabolic rate. Since CysC is produced at a constant rate that is independent of age, gender, muscle mass and external factors like inflammation, they may serve as markers of thyroid function. Indeed, serum CysC levels were found to be significantly correlated with fT4 in hypothyroid and euthyroid status in our study. These findings indicate that serum CysC levels may be a marker of thyroid function. The low levels of CysC in hypothyroidism and the increase in serum CysC levels after L−T4 therapy (12, 13, 14, 17) as well as the significant correlation between fT4 and CysC levels (14) has been also shown in some adult studies. However, changes in serum CysC levels in these studies mostly did not exceed the reference interval. Therefore, it was concluded that serum CysC levels have limited use in determining the peripheral effects of thyroid hormones (17). Similarly, in our study although hypothyroid status lowered CysC levels and L−T4 therapy increased these levels, the fluctuations tended to remain within normal reference interval and CysC levels cannot be accepted as a marker of thyroid status. 

Serum CysC levels did not show a significant difference between boys and girls in the healthy children in our study and this finding is consistent with the results of some studies (6, 8, 9, 24, 25), although gender differences were noted in some other studies (25, 26). In contrast to healthy children, in the patients with hypothyroidism, serum CysC levels were significantly higher in boys than in girls after treatment. Although we do not have a clear explanation for this gender difference in CysC levels in hypothyroidism, we may speculate that thyroid hormones may interact with gonadal hormones in the production of CysC.

There was no correlation between serum CysC levels and age, BMI, height and weight of the children in either the study or the control groups. Serum CysC levels remain constant from the age of 1 year onwards (6, 8, 9, 24) and this offers a considerable advantage in the evaluation of CysC levels in a growing child.

In conclusion, we demonstrated a significant effect of thyroid dysfunction on CysC levels, but the changes in serum CysC levels in hypo− and euthyroid status did not exceed the reference interval. It may be concluded that serum CysC levels have limited use in evaluating the peripheral effects of thyroid hormones.
